# A case of isolated testicular Tuberculosis: Approach and management

**DOI:** 10.1016/j.eucr.2025.103328

**Published:** 2025-12-23

**Authors:** Ismail Mahad Abdullahi, Ismail Ahmed Ali, Abdirahin Mohamed Abdulkadir, Rahma Abdukadir Ahmed, Abdullahi Hassan Elmi

**Affiliations:** Department of General Surgery, Dr. Sumait Hospitals, Faculty of Medicine and Health Sciences, SIMAD University, Mogadishu, Somalia

**Keywords:** Testicular tuberculosis, Hydrocele, Testicular swelling, Caseous granuloma

## Abstract

Testicular tuberculosis is a rare form of genitourinary TB that often mimics conditions such as neoplasms or chronic epididymo-orchitis, making diagnosis challenging. We report a 60-year-old man with longstanding right testicular pain and swelling, no systemic symptoms, and a history of household TB exposure. Imaging showed a large hydrocele with suspected underlying pathology. Intraoperative findings revealed necrotic testicular tissue, prompting orchiectomy. Histopathology confirmed granulomatous inflammation with caseous necrosis consistent with TB. The patient began standard anti-tubercular therapy. This case highlights the need to consider testicular TB in chronic scrotal swelling, particularly in TB-endemic settings.

## 1 Introduction

Tuberculosis (TB) affected an estimated 10.6 million people globally in 2021. Among those impacted, around 6.4 million were men, 3.4 million were women, and 1.2 million were children. This distribution highlights the significant burden TB continues to place on individuals of all ages, with adult males accounting for the largest proportion of reported cases.^1^ Extra-pulmonary tuberculosis (EPTB), which affects parts of the body outside the lungs, made up about 16 % of all TB cases worldwide. Interestingly, this form of TB was more common in the Southeast Asian region, where it accounted for approximately 19 % of the total cases indicating a regional variation in how the disease presents.^2^ Extra-pulmonary tuberculosis (EPTB) occurs when TB spreads beyond the lungs, with urogenital TB affecting the urinary and reproductive systems. This type of TB often develops gradually, and symptoms can differ depending on which organs are involved. It can affect both immunocompetent and immunocompromised individuals. In men, genital TB is considered relatively rare and is usually seen alongside renal TB. When it does occur, it most commonly involves the epididymis or the testes.^1^

Testicular tuberculosis is extremely rare, accounting for only about 3 % of all male genital TB cases. It often presents with symptoms that can mimic other testicular conditions, such as tumors, testicular torsion, or infarction, making accurate diagnosis a clinical challenge.^3^ Diagnosing testicular TB can be particularly challenging, as its symptoms often resemble those of other testicular conditions. To establish an accurate diagnosis, clinicians often rely on imaging modalities such as ultrasound and CT scans to evaluate the extent and characteristics of the lesion. When necessary, ultrasound-guided fine needle aspiration biopsy (FNAB) is performed to obtain tissue samples for further analysis. These procedures are typically supported by laboratory investigations, including culture tests, to confirm the presence of tuberculosis.^1^

This case highlights the diagnostic challenges of isolated testicular TB and emphasizes considering EPTB in chronic testicular masses.

## 2 Case presentation

**Fig. 1:**
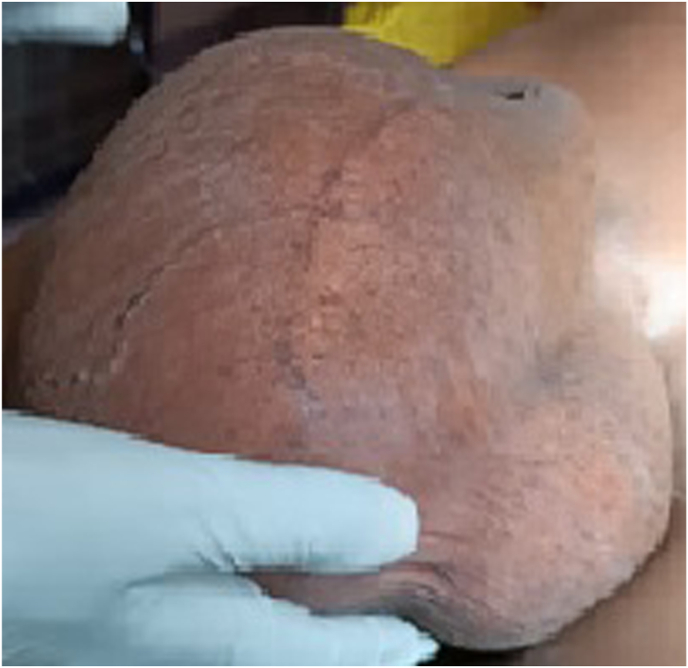
Image of a large testicular mass.

A 60-year-old man with no notable past medical history presented with a six-year history of persistent testicular pain and discomfort. Over the past four years, he also noticed a gradually increasing swelling in the affected testicle. He reported no history of trauma or prior medical evaluation for these symptoms. Notably, his wife had been treated for tuberculosis (TB) two years earlier, which raised clinical suspicion during assessment (Fig. 1).

**Fig. 2:**
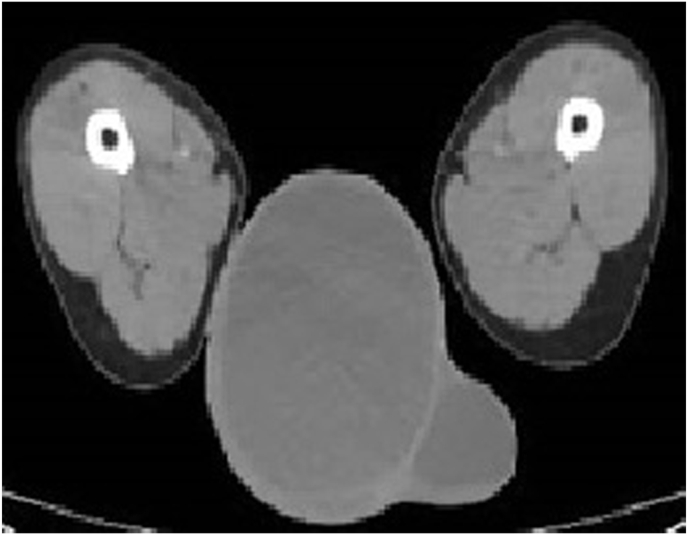
CT scan showing testicular swelling.

On physical examination, a significant swelling was observed in the scrotum, predominantly on the right side. Apart from the scrotal findings, the remainder of the examination was unremarkable. Routine laboratory investigations were within normal limits. Chest radiography didn't show evidence of pulmonary tuberculosis and there were no clinical features to suggest involvement of other extra pulmonary sites. A scrotal ultrasound revealed a large hydrocele, and further evaluation with a CT scan confirmed the presence of extensive hydroceles on both sides, with the right side being more severely affected, measuring approximately 15.47 x 12.48 × 16.80 cm (Fig. 2).

**Fig. 3:**
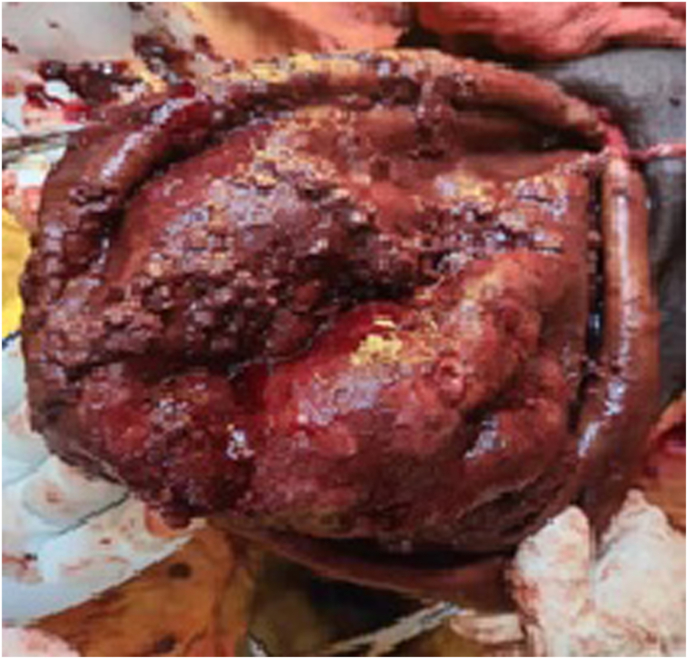
Intra-operative image of the testicle showing caseations.

The patient was initially scheduled to undergo a Jaboulay's procedure to address the hydrocele. However, during surgery, the Surgeon encountered hemorrhagic thick fluid with debris, along with signs of testicular distortion and necrosis. Given these unexpected findings, the surgical plan was revised, and an orchiectomy was performed. The fluid and tissue specimen were sent for Histopathological examination to determine the underlying cause; AFB culture was not performed, (Fig. 3).

Histopathological analysis revealed the presence of well-formed granulomas composed of epithelioid histiocytes and Langerhans-type giant cells, along with areas of caseous necrosis, hallmark features of tuberculosis. Importantly, there was no histological evidence of epididymal involvement, supportingthe diagnosis of isolated testicular tuberculosis.

**Fig. 4:**
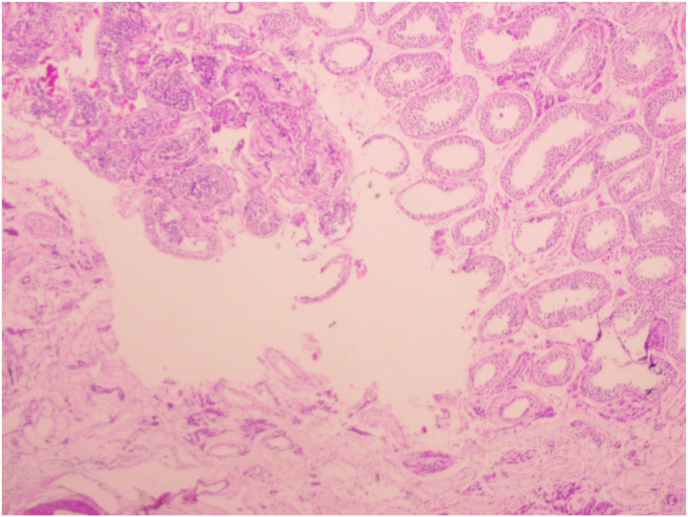
Histopathology picture.

Based on these definitive findings, the patient was started on anti-tubercular therapy in accordance with standard TB treatment guidelines as there were no clinical features to raise concern for drug-resistant disease and his spouse improved with 6months of standard treatment regimen. On follow-up, he showed good postoperative recovery, with improvement in local symptoms and no signs of disease progression (Fig. 4).

## 3 Discussion

Genitourinary tuberculosis (GUTB) accounts for roughly 27 % of all extra-pulmonary TB cases, making it the second most common form after lymph node involvement. It typically begins in the kidneys and can spread to the ureters and bladder. Involvement of the external genital organs is relatively rare. Isolated testicular TB is particularly uncommon, representing only 2–3 % of GUTB cases, and it is often seen alongside infection of the epididymis or prostate.^4^ The global rise in tuberculosis cases has been significantly influenced by the spread of AIDS, the emergence of multi-drug resistant TB, and increased rates of international migration. This surge has been especially pronounced in African countries, where the burden of the disease continues to grow.^5^^,^^6^

The exact pathway by which tubercle bacilli reach the scrotal structures remains a subject of debate. However, in most cases, TB epididymo-orchitis is believed to occur through the retrograde spread of bacteria from an infected urinary tract. The bacilli are thought to travel backward into the prostate via urinary reflux, and then extend through the reproductive tract, involving the seminal vesicles, vas deferens, and eventually the epididymis. While the bacteria can also spread through the bloodstream or lymphatic system, this is less common. Testicular TB usually arises from local extension or retrograde seeding from an infected epididymis. Therefore, TB of the testis without epididymal involvement is considered extremely rare.^7^

Scrotal tuberculosis, or TB epididymo-orchitis, can affect one or both sides of the scrotum—appearing unilaterally in about 66 % of cases and bilaterally in 34 %. It may present with either acute or chronic scrotal swelling, which can be painful or painless. In many cases, the swelling is accompanied by inflammation and swelling of the overlying scrotal skin. Clinical signs that raise suspicion for scrotal TB include a non-tender testicular mass, a firm and enlarged epididymis that is also non-tender, thickened or beaded vas deferens, and noticeable scrotal edema. A markedly swollen epididymis is often due to chronic granulomatous inflammation and blockage of the vas deferens. These findings can closely mimic those of testicular tumors, making diagnosis more challenging. One distinctive feature of TB involvement is a beaded appearance of the vas deferens caused by granulomatous changes. In more advanced cases, scrotal fistulas or sinuses may form, often draining thin, odorless pus—a finding that strongly points toward tuberculosis as the underlying cause.^2^

Tuberculosis affecting the male reproductive system can result in infertility, mainly due to inflammation and scarring that disrupt normal anatomical structures and block the reproductive pathways. In some cases, infertility may actually be the first noticeable sign of genitourinary tuberculosis, with patients often unaware of having experienced any prior symptoms.^8^

Ultrasound is the primary imaging tool used to diagnose testicular tuberculosis, with a sensitivity of over 80 %. Typical ultrasound findings include a heterogeneous appearance of the testicular tissue, along with areas of necrosis that appear darker (hypoechoic) and, in many cases, the presence of calcifications—seen in about 50–60 % of patients. Doppler ultrasound can help distinguish TB from testicular cancer by showing a lack of blood flow in the necrotic areas, which is less common in malignancies. Other helpful signs include thickening of the scrotal wall and involvement of the epididymis, both of which are seen in roughly 60–70 % of cases. These features play a crucial role in identifying and confirming testicular TB.^1^

CT and MRI are useful tools for evaluating scrotal masses and can help distinguish testicular tuberculosis from other conditions. On CT scans, testicular TB typically appears as poorly defined, heterogeneous lesions, often showing ring-like (annular) or multilocular patterns of enhancement. MRI, with its superior soft-tissue contrast, is particularly helpful in precisely locating and characterizing scrotal abnormalities. While these imaging techniques provide valuable information, there is currently no specific data available regarding their sensitivity and specificity in diagnosing testicular TB. Nonetheless, they play an important role in supporting the clinical diagnosis and guiding further management.^4^

Ultrasound-guided fine needle aspiration biopsy (FNAB), followed by histopathological examination, is considered the gold standard for diagnosing testicular tuberculosis. This method typically reveals caseating granulomas with a central area of necrosis. In 30–50 % of cases, acid-fast bacilli can also be detected using Ziehl-Neelsen staining, further confirming the diagnosis. The presence of granulomatous inflammation with caseation is highly specific to tuberculosis. However, in some cases, non-caseating granulomas may be seen, which can also occur in other conditions like Sarcoidosis or idiopathic granulomatous orchitis. In such instances, it's difficult to definitively rule out TB without additional microbiological evidence.^1^

According to WHO guidelines, the standard treatment for testicular tuberculosis involves a six-month course of anti-tuberculosis medication. This includes a combination of Rifampicin, Isoniazid, Pyrazinamide, and Ethambutol for the first two months, followed by Rifampicin and Isoniazid for the remaining four months. This regimen has been shown to be highly effective in completely resolving testicular TB lesions when followed properly.^3^

In a study by Huang, Y et al., surgical intervention for patients with tuberculous epididymo-orchitis (TBEO) was recommended under specific circumstances. These included cases where 1–2 months of standard anti-tuberculosis treatment failed to adequately control the infection, or when the disease had progressed to an advanced stage with widespread spread of *Mycobacterium tuberculosis*. Surgery was also considered necessary in the presence of complications such as hydrocele, abscess formation, sinus tracts, or fistulas. Additionally, if there was a strong clinical suspicion of a scrotal tumor needing further evaluation, or if the patient was initially diagnosed with nonspecific bacterial epididymo-orchitis that did not respond well to antibiotics, surgical management was advised.^4^^,^^6^

## 4 Conclusion

This case highlights the need to consider testicular tuberculosis as part of the differential diagnosis for chronic scrotal swelling, particularly in regions where TB is common or when there are known risk factors, such as close contact with someone who has TB. A thorough diagnostic workup including histopathological examination and timely initiation of anti-tubercular treatment are crucial for achieving the best possible outcomes in this uncommon form of tuberculosis.

## CRediT authorship contribution statement

**Ismail Mahad Abdullahi:** Writing – original draft, Data curation. **Ismail Ahmed Ali:** Writing – review & editing, Supervision, Conceptualization. **Abdirahin Mohamed Abdulkadir:** Writing – original draft. **Rahma Abdukadir Ahmed:** Writing – review & editing. **Abdullahi Hassan Elmi:** Writing – review & editing.

## Ethics and consent

A Written consent form was attained from the patient for publication of this case report and the attached images. In our institution, ethical approval is not required for case reports.

## Declaration of generative AI use

During the preparation of this work, the authors used ChatGPT to support language clarity and enhance readability. Following its use, the authors carefully reviewed and edited the content to ensure accuracy and coherence, and take full responsibility for the final version of the manuscript.

## Funding source

The authors received no financial support for the research, authorship, and/or publication of this article.
